# Biological constraints as norms in evolution

**DOI:** 10.1007/s40656-022-00483-1

**Published:** 2022-03-03

**Authors:** Mathilde Tahar

**Affiliations:** ERRAPHIS, Université Toulouse II - Jean Jaurès, Toulouse, France

**Keywords:** Theory of evolution, Biological constraints, Possibility space, Normativity, Biological agency, Historicity

## Abstract

Biology seems to present local and transitory regularities rather than immutable laws. To account for these historically constituted regularities and to distinguish them from mathematical invariants, Montévil and Mossio (Journal of Theoretical Biology 372:179–191, 2015) have proposed to speak of constraints. In this article we analyse the causal power of these constraints in the evolution of biodiversity, i.e., their positivity, but also the modality of their action on the directions taken by evolution. We argue that to fully account for the causal power of these constraints on evolution, they must be thought of in terms of *normativity.* In this way, we want to highlight two characteristics of the evolutionary constraints. The first, already emphasised as reported by Gould (The structure of evolutionary theory, Harvard University Press, 2002), is that these constraints are both produced by and producing biological evolution and that this circular causation creates true novelties. The second is that this specific causality, which generates unpredictability in evolution, stems not only from the historicity of biological constraints, but also from their internalisation through the practices of living beings.

## Introduction

The evolution of biological forms and functions does not seem fully explained by the laws that make physics intelligible (Longo et al., 2012). Evolution is less a mechanical sequence based on efficient causality than a process whose entities are in perpetual change (Dupré & Nicholson, 2018; Simons, 2018). It displays a continuity of forms, but also a progress: the lineages evolve in certain directions, without the space of the possible directions being predetermined. This does not mean that there is no stability nor regularity in the evolutionary process. On the contrary, evolution appears to be highly channelled. To account for biological regularities, Montévil and Mossio have proposed a theory of *constraints* (2015). This concept aims to explain how a certain entity can act, at a given moment, on processes. These entities are constraints for specific processes: they are local. They are also transient because historically constituted (Montévil, 2020). Through this concept, the authors manage to explain the relative stability of biological systems as a closure of constraints that channel biological processes. In this article, we will question the role of these constraints in evolution, beyond the scale of biological systems. Our aim is to account for their emergence from evolutionary history and to understand how they explain, and how they canalise the directions taken by evolution. We are therefore less interested in understanding how they stabilise biological systems than in identifying the causality of these constraints, which makes them a real driving force in evolution and generates biodiversity.

The argument of this article is that biological constraints should be thought of as norms when trying to appreciate their causal role in the evolution of the biodiversity structure. It has been argued (Longo et al., 2012) that constraints in evolution are not necessitating their effects but enabling certain evolutionary directions. In this article, we wish to take up this idea by analysing more precisely the causal force of these constraints in terms of normativity, in order to give a concrete account of causation in evolution.

## Trying to account for the regularities of evolution

Evolution appears to be a relatively determined and channelled process. It displays a certain coherent stability: the evolution undergone by a species appears as linear and progressive; we observe similarities between organs performing the same function in different lineages. It also seems that not all transformations are possible; some morphologies may be inaccessible to some species: as Fodor (2007) says, pigs will probably never have wings. But although there is now a solid theory of evolution based on Darwinism and genetics, it remains difficult to comprehensively account for these regularities and to fully identify their sources.

### *Τelos* in evolution?

In order to explain evolutionary regularities, attempts have sometimes been made to use the Aristotelian concept of *telos*. Dennett for instance conceives of evolution as the progressive actualisation of the different possible phenotypes within a pregiven “Design Space”: “Design is Aristotle’s *telos*, an exploitation of Order for a purpose” (1995, p. 64), the purpose of an organ adapted to a given ecological problem. What would explain the regularity of evolution would be the limited number of available solutions, i.e., of possible phenotypes. Beyond the shortcomings of such a teleological approach of evolution (Grene, 1972), the main problem with this conception is that it implies a form of fixism or essentialism, which would consist in thinking that all the possible biological forms would be given a priori, from all eternity, evolution being just the history of their materialization. This leads us back to an idealistic vision, of the same type that Darwin already refuted, which made the species a form existing from all time. As Darwin showed, species is a naturalist’s abstraction, the species being merely more pronounced varieties, the evolution of a lineage consisting of the gradual transformation of an individual difference into a variety, then a species (Darwin, 2001, p. 54; pp. 60–61). There are no pre-ordained forms, prior to evolution.

### The library of mendel

Another way of grasping the regularities in evolution (complementary to the first) would be to assume that there would be rules preceding evolution, given by genetics. This idea is present in Dennett (1995) who speaks of a “Library of Mendel” as a genetic repertoire latently indicating the possible directions of evolution. It is true that genes structure evolution insofar as they carry the information allowing the formation of the organism, and a certain number of genes or groups of genes are found in different lineages—such is the case of the *Hox* gene which direct the development of the antero-posterior axis in vertebrates. But genetic sets change from one species to another, and from one organism to another, and more importantly the genetic processes themselves evolve over time. For instance, the presence of the *Pax-6* gene (which is also a development gene but structurally distinct from the *Hox* gene) in several phyla (not only is it present in cephalopods and humans, but homologous genes are found in drosophila and fish) seems to reveal a strong necessity in the eye’s induction processes. But its functioning and expression are specific to each species: it controls the action of other genes which are specific to different species and whose effect is to result in eyes which are also specific. “[…T]he mechanism of eye induction may be conserved across the animal kingdom. However, [there is a] dramatic variation of eye structure not only between vertebrates and invertebrates, but also within the vertebrate lineage” (Neumann & Nüsslein-Volhard, 2000, pp. 2138–2139). The way in which genes are expressed is not preordained and depends on the unique biological situation of the organism, which results from evolution itself.

### Natural selection and the adaptation requirement

To account for evolutionary regularities, some have proposed to conceive of natural selection as an algorithm that would sort through the genetic repertoire. According to Dawkins (1996, pp. 46–50), this algorithm should be thought of as follows. A text would be typed randomly and copied several times with a certain degree of random errors. The computer would choose, among the descending sentences of this first set of random letters, the one that would most resemble a “target phrase” determined in advance: the closer the phrases would be to the target, the more they would reproduce. The action of natural selection would be comparable to that of this algorithm which eliminates the texts furthest from the target; in evolution, the target would be adaptation. Even if it takes different forms depending on the situation, adaptation would be maintained as the general form of an imperative: that of survival and reproduction. Indeed, lineages persist by virtue of the interactions that organisms have with the environment, and these interactions are only maintained if they guarantee an adjustment of the species to the ecosystem in which they evolve. Therefore, adaptation could be considered as the consistent rule of evolution, explained by natural selection and that would account for the directions taken by the evolutionary process. However, this rule appears to be more of an imperative of efficiency (an individual who is not able to survive… does not survive), a minimal condition, than a real principle of explanation of the form taken by biodiversity and morphological evolution.

### Failure of these proposals

All these attempts to account for the regularities in evolution derive from a static, nomological view of evolution as resulting from immutable laws. This is why they try to account for the observed stabilities in a monolithic way, even if it means falling into a deflationary explanation that only gives a negative account of the directions taken by evolution. Jointly, the entities under consideration (organisms, species) are seen as ontologically preceding evolution. This is a substantialist view of evolution, outdated since Darwin. Evolution is the process by which species are generated and evolve (let us note that it is species or populations that evolve, not individuals); from this point of view, both species and organisms are secondary to evolution. They take their forms from this process, and, above all, they are transient in respect of the process itself. Biological evolution is indeed a *process* in the strong sense given by Dupré and Nicholson (2018, pp. 11–14): it is extended in time, and this flow of time means change. Change is thus ontologically primary with respect to the entities that it produces. Thus, in biology, “entities” appear as “transient patterns of stability” (*Ibid.*, p. 13): organisms are constantly changing, species evolve, diverge, become extinct, new species arise… They are not *fixed* entities, but processes that are relatively stable (Miquel & Hwang, 2016), or, as Peter Simons puts it “precipitates of processes” (Simons, 2018, p. 55). What is fundamental in evolution is not the entities, which are always transitory, but the dynamic processes that give rise to their relative stability (Soto et al., 2016a, 2016b).

Thus, in order to explain the regularities in evolution, it is necessary to move away from a nomological and monolithic approach and to take into consideration their historicity.

## Evolutionary constraints and history

### Historical constraints

Following Gould and Lewontin (1979), we do not deny either the necessity of adaptation nor the constraint of the genetic heritage but wish to demonstrate that these conditions alone are not enough to explain the diversity of forms or the dynamics of the process. Evolution towards adaptation is subject to multiple other conditions, which, as Gould shows (2002, pp. 1027–1037), appear as constraints for a pan-adaptive explanation; since they are non-classical factors that act differently from what orthodoxy predicts. But they are also real constraints since they canalise change, whether they are structures inherited from the past or physical determinisms. These constraints should not be understood in the sense of mere obstacles, but also in their positivity. Gould posits two characteristics of constraint (2002, p. 1026):

1. “Coherent set of causal factors that can promote evolutionary change”.^1^
2. “The concept of constraint must include theoretically legitimate and factually important *positive* meanings—i.e., constraints as *directing causes* of particular *evolutionary changes*—rather than only the negative connotations of structural limitations that prevent natural selection from crafting an alteration that would otherwise be favored and achieved”.

Gould distinguishes three types of constraint, all of which are linked to the history of organisms and thus also of evolution. The first is the *functional* constraint, which comes from natural selection, i.e., the necessity of adaptation, the terms of which are set by the current conditions of the environment. It must be stressed that even this constraint has a historical dimension insofar as the environment sorting out the fitted variations is also shaped by the way organisms have lived in and thus transformed this environment. In addition to this first “orthodox” constraint, there is the *structural* one, which can come from the direct action of physical laws on the plastic matter (an ahistorical phenomenon)—for instance, the fact that flying fish fall back into the water because of their mass, is not only a physical necessity but a biological aptation (*i.e.,* it is *aptive*, even though it is not an *adaptation* built by natural selection) (2002, p. 1054). But it can also be the constraint of the architectural laws determining the appearance of traits as non-adaptive corollaries of other structures, which can then be co-opted. Gould gives as an example the correlation between the presence of a second row of teeth and the abnormal hairiness of Julia Pastrana (*Ibid.*, p. 1055). This correlation is determined by homological constraints between hairiness and dentition that originate in the phyletic singularity of mammalian development. These constraints are born of the evolutionary history of organisms, but they can also guide future evolution by canalizing evolutionary change. This is the case of the fontanelles, constraints specific to vertebrates, due to the development of the skeleton, and which subsequently became functional in mammals insofar as they allow the skull to deform during the compression of parturition (Darwin, 2001, p. 180). Finally, there are the *genealogical* constraints: traits inherited from ancestral forms, whether or not it has been shaped by natural selection, which constrain and positively orient both the possibilities of immediate change and the settlement of the morpho-space.

**Fig. 1:**
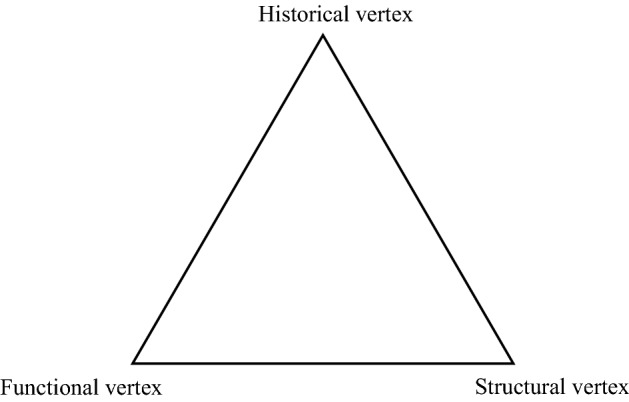
Aptive triangle redesigned from Gould, (2002, p. 1052). The three vertices correspond to the different influences on the formation of a trait (the trait will correspond to a point located inside the triangle according to the respective influence of these three vertices). “Standard triangular diagrams for depicting basic causes of form as functional (immediate adaptation to current circumstances), historical (inherited by homology, whatever the basis of ancestral origin), and structural, or arising either as physical consequence of other features or directly from the nature of physical forces acting on biological materials. All vertices may yield aptive traits of great utility to the organism” (Gould, 2002, p. 1052)

To account for the positive dimension of these constraints, Gould thinks of their role in the formation of *aptive* traits. He proposes an “aptive triangle” based on the model of the triangle used in petrology (Fig. 1). Each of the vertices corresponds to one of the three constraints, understood as an influence canalizing evolution. To explain the appearance of a trait, all the three vertices should be considered (the trait will correspond to a point located inside the triangle according to the respective influence of these three idealised vertices). However, if the condition of evolution is the adaptation of organisms, an adaptation subject to internal constraints, what seems fundamental to understanding the respective role of these constraints in the appearance of a trait and thus, more broadly, the directions taken by evolution, is history. For it is history that explains the inhomogeneous distributions of organisms across the potential morpho-space of good organic design. The occupation of morpho-space is not explained by natural selection alone: it does not represent the figure of the best possible solutions to functional problems, but depends on a bundle of constraints, which are all ultimately explained by history (Gould, 2002, pp. 1055–1056).

### The ever-changing space of possibilities

This conception makes it possible to account for the plurality of evolutionary constraints, as well as for their historicity. Nevertheless, their action is not only negative: they channel the future by opening its possibilities. If constraints were completely stable, the evolutionary process would only be entropic, and thus predictable: as time progresses, organisms would occupy more and more possible places in morpho-space; a decrease in organisation and an increase in disorder would be achieved. Yet, what is observed is “a decisively non-random, occupation of a theoretical ‘design space’” (Gould, 2002, p. 1055), and even *an increasing organisation in the genealogies and in the organisms* (based on the observation that living forms become more diverse and complex, Bailly & Longo, 2009; McShea & Brandon, 2010) *which is opposed to the idea of entropy itself*. This problem is taken up by Brooks and Wiley (1988). According to them, evolution *is* an entropic phenomenon (because of the variability of organisms, the further time goes by, the more possible genotypes, i.e., possible microstates, are occupied by organisms), *even though* a growing organisation in biodiversity is observed, i.e., an increasing distance from randomness, both within organisms and in the structuring of niches. To understand this, the historical dimension of evolution and its constraints must be considered.

**Fig. 2:**
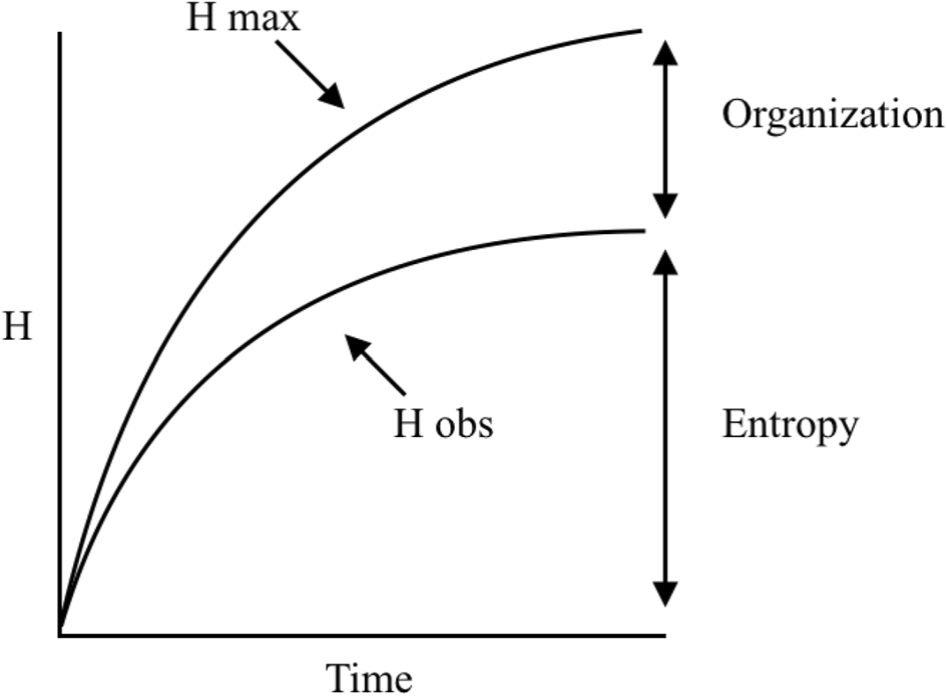
The entropy in evolution, redesigned from Brooks & Wiley, (1988, p. 40). The difference between *H*_*max*_ (increasing entropy maximum) and *H*_*obs*_ (the observed entropy) is organization, while the value of *Hobs* is a measure of the entropy of the system. If this scheme is applied to evolutionary lineages (1988, p. 43), *H*_*obs*_ measures the historically realized diversity. The historical exclusion of the expression of certain kinds of information is measured by the difference between *H*_*max*_ and *H*_*obs*_. The aera above *H*_*max*_ corresponds to impossible combinations because no novelties exist (at the time observed). But what is impossible at one moment may become possible at a later time period. It can also be applied to represent the gap between the number of possible genotypes *H*_*max*_ and the distribution of observed genotypes *H*_*obs*_ (1988, pp. 44–45)

In biology, unlike in physics, history has a special efficacy in that it is not the exploration of a pregiven space of possibilities (or phase space): the phase space is constantly changing in ways that cannot be predicted. Brooks and Wiley conceive this change in terms of growth: the more time goes by, the more microstates there would be. Organisation would emerge from a difference in rhythm: the increase in the phase space would be *faster* than the realised increase in entropy (Fig. 2). Longo, Montévil and Kauffman show that in reality the change in phase space is not quantitative. Since the relevant observables in biology are not invariants (they are instead characterised by their variability, Longo et al. ) and since the constraints that structure the phase space are themselves historical (Montévil, 2020), *it is the very structure of the phase space that changes over time,* albeit with a coordinated increase in entropy and organisation. This last point stems from the fact that the phase space itself is historically constituted. It transforms while retaining something of its past structure: the ontogenetic constraints, born of evolution, canalise the successful realisation of genotypes, even if the genotypes not realised may be accessible microstates; certain genotypic combinations are also made impossible by the fact that speciation has distributed genetic information across different lineages over time (lineages that do not reproduce each other).

Therefore, it is the *historical* dimension of constraints that gives them their *positive dynamic*. Constraints in evolution emerge from the evolutionary process and orient it by redefining the space of possibilities. Not only do they canalise change as they prevent biological systems from occupying all possible genetic configurations, but *they themselves evolve in such a way that the space of morphological phases is constantly changing*: they create new possibilities, which will become new constraints (Montévil, 2019). It is this creative dimension that must now be investigated.

## Biological interactions and evolutionary norms

To understand how these constraints can emerge from evolution while canalising it, it is necessary to grasp the very process of evolution in its concreteness. Dupré and Nicholson define it, in the same way as rain, light or fermentation, as an unowned process (2018, p. 12), because the entities of this process are not its substrates but its precipitates. Yet, in evolution, the precipitates are at the same time the transitory agents of this process, for evolution is the history of the interactions of living beings. As Fisher writes, the motor of evolutionary change is “in the actual life of living things; in their contacts and conflicts with their environments, with the outer world as it is to them; in their unconscious efforts to grow, or their more conscious efforts to move. Especially, in the vital drama of the success or failure of each of their enterprises” (1950, p. 184). Thus, it could be said that since evolution belongs to no one in particular, it belongs to everyone: it is a process knotted by interactions whose agents are in in(de)finite number, that is to say knotted by an indefinite number of processes (the agents being, as we have seen, stabilised processes).

### Constraints and interactions

Indeed, if the horizon of evolution is the adaptation of organisms to their environment, this constraint is dynamic: it comes from a history and shapes this history. It is not a constraint imposed unilaterally and externally by the environment but is constituted by the history of the interactions of organisms with their environment, as they also transform this environment. The causality is not unilinear. It cannot be said that the niche causes the means of survival of the living being and its evolution, since the niche is what allows the living being to survive (by definition, otherwise it would not evolve in this precise environment). The phenotype of a living being, and its niche are defined by each other; hence, no causal priority can be given to one or the other (Fodor & Piattelli-Palmarini, 2011). Niches are not waiting to be filled until the day when miraculously the right phenotype appears. *The evolution of the forms of living beings cannot be understood as responses to the constraints set by the environment, since environment and its stakes are defined on the basis of phenotypic traits that are supposed to be adaptations*: “But that there are spiders, who would have guessed how to spin webs to catch flies is an ecological problem?” (*Ibid.,* p. 140). Fodor and Piattelli-Palmarini denounce an inverted reasoning of the type: locks exist for keys to open them, rather than keys are made to open locks. They answer the question of this chapter “Did the dodo lose its ecological niche? Or was it the other way around?”: “The extinction of the dodo was the very same event as the extinction of the dodo’s way of making a living so neither can serve to explain the other” (*Ibid.,* p. 147). The constraint emerges from the horizon represented by the niche: it is neither given by the environment unilaterally nor by organisms.

It is the history of the co-construction of the niche that supplies the conditions for the survival of organisms and species, thereby channelling the evolutionary process in certain directions that will produce the new constraints of the niche. Thus, these constraints are the products of interactions, i.e., concrete, practical relations, which involve agents: the living beings in relation with each other and with their environment. Living beings are not (necessarily) cognitive agents, but they are agents insofar as they actively take part, through their interactions, in evolutionary history. “An updated account of evolution […] should, at least, represent adaptive evolution […] as the interaction of agents and their affordances. It should take seriously the Darwinian insight that evolution is the direct consequence of what organisms do” (Walsh, 2015, p. 241). Hence, constraints in evolution should rather be thought of as *norms.*

### Normative interactions

This does not mean that it is the agents who directly behave normatively, but that *their interactions have a normative part.* To capture this, Brooks and Wiley use the image of a playground (1988, p. 83). Organisms impose constraints which come from their genealogy and define the conditions of their self-organisation. There is also an “ecological hierarchy” which refers to the relationship between organisms and their environment and which defines another type of constraint: boundary conditions. The latter give the playing field, where the constraints of the organisms produce the rules of the game. Several games can be played on the same field, but only one at a time. Biological systems therefore obey organisational rules, and their playground is the boundaries defined by the planet. Evolution is the meeting of changes in the rules of the game and of correlative changes in the dimensions of the playing field, inasmuch as these changes are reflected in the biological interactions of organisms.

So far, we have discussed the interaction of organisms with their environment, but evolution depends on multiple interactions that go far beyond this bilateral relationship. As Darwin himself pointed out, it relies on the intensity of the struggle for existence, which is not only a struggle with the environment, but a struggle between individual agents for access to resources that can take very various forms. Although we cannot exhaustively illustrate the complexity of the interactive processes at work in evolution (which range from competition between cells in development to the differentiated extinction of species in catastrophic upheavals), we will nevertheless give an example: that of competition between species.

### Interactions of wolbachia

One aspect of this competition is the relationship between the parasite and its host. This relationship has a normative dimension in that it channels the evolution of the two species involved, but this channelling is highly dependent on the way the biological agents interact in their unique biological situation. The interaction of the bacterium *Wolbachia* with different insects is a remarkable example. In the hymenopter *Asobara tabida*, *Wolbachia* is indispensable for the ovogenesis of its host (Dedeine et al., 2001). But in most other insects, *Wolbachia* is a reproduction parasite. In *Hypolimnas bolina*, it kills males in the embryonic stage, conserving only females (Dyson & Hurst, 2004). In the woodlouse *Armadillidium vulgare*, *Wolbachia* doesn’t kill males but induces the development of woodlouse embryos into females, irrespective of the sex chromosomes carried by the embryos (Cordaux et al., 2011). In *Armadillidium vulgare*, there is chromosomal sex determination based on female ZW/ZZ heterogamety. But when genetically male embryos carry *Wolbachia*, they develop into functional females. *Wolbachia* thus clearly increases its reproduction since instead of being in a male which will not transmit it to any of its descendants, it is in a female which will transmit it to almost all its descendants. In addition, *Wolbachia* can pass on its *f* genetical factor which feminises males (once *f* is passed on, there is no need for *Wolbachia* to be present for feminisation). Some populations of ZZ females can even be found without the presence of the *Wolbachia* bacterium (Leclercq, et al., 2016). However, the nuclear genes carried by the majority sex in the population are disadvantaged. The risk is that there will be no more male, and therefore no further reproduction of the woodlouse. As a result, genetic resistances to feminisation have been observed in natural populations of *Armadillidium vulgare*: mainly the M gene which is able to restore the male phenotype in the presence of *f* (Rigaud & Juchault, 1993); but also, a polygenic system that has no direct impact on the phenotype but limits the rate of transmission of *Wolbachia* to the offspring (Rigaud & Juchault, 1992). Another form of resistance, also present in *Hypolimnas bolina* (Charlat et al., 2007), is the development of a strong copulatory capacity in woodlouse males (Moreau & Rigaud, 2003). Interactions with *Wolbachia* have very different evolutionary consequences from one species to another, but also within populations of the same species. It should also be underlined that the resistances are passed on from one generation to the next. The interaction has therefore created new constraints in the long term, through genetic changes. The constraint of adaptation here translates into the fact that the woodlouse must evolve to survive (it is a good example of the Red Queen hypothesis, Van Valen, 1973). The genetic constraint is also involved: evolution depends on the genetic variability available. But what really allows these constraints to orient evolution and transform the process from which they emerge, is the normativity of the interaction itself, as it takes different forms from one species to another, and from one population to another.

This normativity of interactions is crucial in medical biology to understand diseases (see Méthot & Alizon, 2015). The virulence of a microbe, for instance, depends on the specific interaction between the pathogen and its host: a microbe is not intrinsically pathogenic, it is the relationship with its host that will define whether it is pathogenic. In addition, some microbes, initially non-pathogenic on a particular host, may subsequently evolve and become pathogenic to that host. This property of pathogenic or not, which seems to be a normative property *par excellence* ‘good’ or ‘bad’, depends on the host, the microbe, and their interaction. It is by understanding this normative relationship that we can grasp the direction taken by evolution. If the parasite is not pathogenic, then there can be co-evolution with its host; if it is weakly pathogenic, there can be an ‘arms race’; if it is highly pathogenic, this can lead to the extinction of the host species.

These interactions also define their own evolutionary norms in relation to their environment. Let’s consider two species of birds from different lineages. The first, due to its evolutionary history, can only feed on berries while the second can feed on both berries and seeds. Suppose that the environment contains berries and seeds in equal proportion: it can be assumed that the species that feeds on both berries and seeds will feed on the seeds, so as not to compete with the other species. Here there are evolutionary processes coming from speciation that meet and structure the ecological space. In return, this patterning has a retroactive effect on the evolution of these two species: the second species may come to lose its ability to ingest berries. It is in the reciprocal influence of constrained processes that evolutionary normativity emerges.

Thus, the role of biological constraints in the directions taken by evolution depends on the diversity of organisms and their particular ecological situation. *It is these various concrete interactions that explain the creativity of constraints, and which make it necessary to speak, in the case of evolution, of evolutionary norms.*

### Evolutionary norms

The word norm is used in specific cases in biology: the normal versus the pathological in medical biology (a polarity famously conceptualised by Canguilhem, 1966), or in the established expression “norms of reaction” dealing with phenotypic plasticity (West-Eberhard, 2003; Woltereck, 1909). We speak of norm in a broader sense, since it is the very causality at work in evolution that we propose to think of as normative. In order to draw up an operational definition of the concept for evolutionary biology, let us review its different meanings. Apart from the strictly mathematical sense, we identify three main meanings, based on the definitions listed by Merriam-Webster Dictionary and ATILF Lorraine (which relates to the French word, but is more detailed):

- A regular state in relation to an average.
- A state of conformity in relation to an average, which then takes on the value of an “ideal type”.
- A standard to which one refers to judge, but also to act. Thus, in human productions, the norm takes on the meaning of a “rule, law in an artistic, scientific or technical field; conditions that must be respected in an accomplishment; prescription that must be followed in the study of a science, the practice of an activity, an art” (ATILF—CNRS & Université de Lorraine, our translation). It is not a rule only for judgment, but for practice. Another important characteristic is that it depends on the socio-cultural context, which is itself changing; it emerges from practices. This definition emphasises the dynamic dimension of the norm: it gives directions to a practice, and it emerges from a particular context which comes from the very history of this practice: it is therefore historical or evolutionary. The norm has both a creative positivity (its power does not only consist in a limitation but also in the fact that it constitutes opportunities) and emerges from and structures the interactions and practices of agents who live these norms.

(a) and (b) are static definitions, and therefore are not adequate for thinking of evolution. It is from (c) that we think we should speak of norms in the evolutionary process although leaving aside the cultural framework. These norms are:

1. Transitory rules which are,
2. Both produced by and producing a historical process, and
3. Have a creative dimension (they do not only limit or constrain the process, but transform the space of possibilities)
4. Deriving from the fact that they are internalised in interactions, i.e., practices giving them their power and normative meaning.

Grasping this normativity of biological constraints in evolution also allows us to understand their causal modality, beyond traditional logic and a substantialist conception of evolution. It is not just a matter of replacing one word with another: constraints in evolution *do have a normative power*.

## The normative power of evolutionary constraints

### Their positive value

Biological constraints are not necessitating causes (Longo et al., 2012): they do not cause a predetermined effect. Nor can they be said to actualise possibilities, since as we have seen, the possible is not predefined in biology. They form opportunities for evolution itself (see also Jacob, 1977 or Caporael et al., 2014), opportunities that were not predicted, and which generate real novelties.

This is why Montévil speaks of constraints as possible innovations: “the constraint plays a role, and this role is performed in a specific manner by generating other constraints” (Montévil, 2019, p. 4574). He takes the following example: “articulated jaws enabled teeth such as molars which can crush food. However, crushing food with the mouth was and still is not an actual possibility at all for Chordates without articulated jaws” (*Ibid.,* p. 4575). The evolutionary novelty of articulated jaws is part of the organisation of an organism in such a way that it becomes a structural constraint, but also widens the space of possibilities in the organism by offering the opportunity for a new function: crushing food. The emergence of the first novelty (articulated jaws) is a necessary ingredient for the second novelty (teeth) to be able to play a functional role (crushing food). Thus, new traits allow secondary novelties that could not have been foreseen before the appearance of these new traits. And it is by this very fact that innovations acquire their constraining aspect in the sense of a stabiliser: “a novelty may become deeply integrated into certain biological organizations, making its complete disappearance unlikely to be viable” (*Ibid.*, p. 4576). Both a channelling force and a breeding ground for innovation, evolutionary constraints have both aspects (1) and (2) of norms. We may add from the example we have just studied that it also has a creative dimension (3).

### The creative circularity of constraints

**Fig. 3:**
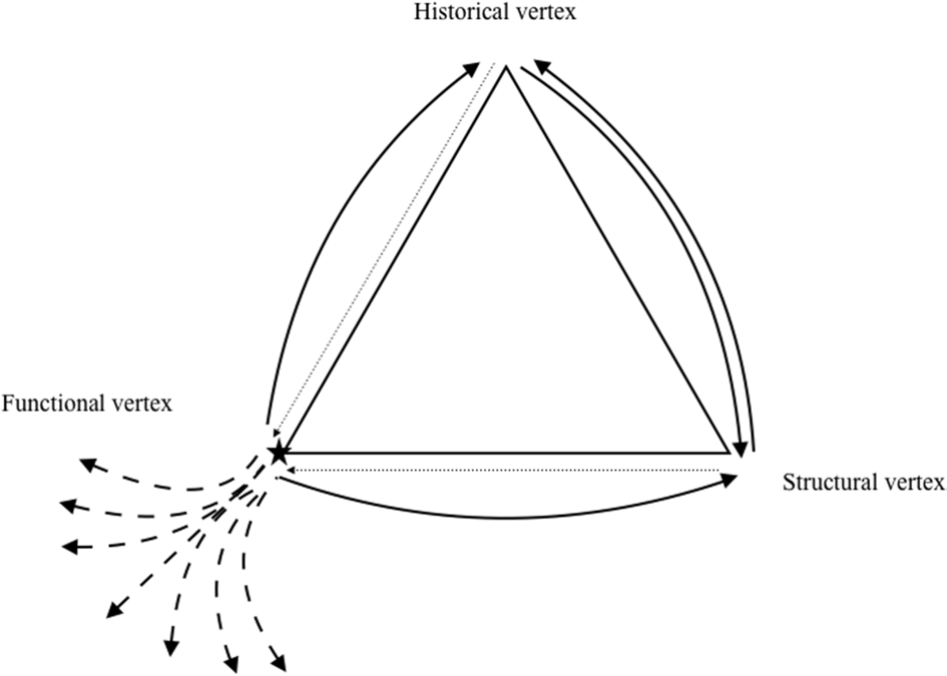
Reinterpretation of the aptive triangle from Gould, 2002, p. 1059. The arrows  reflect the functionalist interpretation of the theory of evolution, which sees history and structure as constraints imposed on the functional vertex. The arrows  are added to highlight the causal circularity of constraints on each other. The arrows  indicate the various meanings that these constraints can take on for the organism. They usually appear successively. They may be contemporary with each other (functional overload), or on the contrary be mutually exclusive (exaptation). These different meanings are not predictable because they arise from the causal circularity of the constraints which are themselves transitory

But to better appreciate (3), let us consider a more paradigmatic example found in Gould, which highlights the creativity of constraints by revealing the diversity of meanings they can take on in the course of history. This example follows on from an excerpt of *The Genealogy of Morals* by Nietzsche on historical research, that Gould (2002, p. 1216) uses to think of evolution: “‘Namely, that the origin or the emergence of a thing and its ultimate usefulness, its practical application and incorporation into a system of ends, are *toto coelo* [entirely, or literally ‘to the highest heavens’] separate; that anything in existence, having somehow come about, is continually interpreted anew, requisitioned anew, transformed and directed to a new purpose’”. Rather than simply referring to cases of exaptation (a change in function of a structure in the course of evolution), Gould gives an example of what could be called an “*overloading*” (Longo, 2018, pp. 457–458) which further reveals the abundant and unpredictable creativity of evolutionary constraints. While Gould does not embrace the full Nietzschean analysis, he “do[es] appreciate Nietzsche’s point, which can be translated into evolutionary terms as the source of constraint” (Gould, 2002, p. 1218). He adds: “The original reason does continue to exert a hold upon history through the structural constraints that channel later usages. Once feathers originate for thermoregulation, the form of any later utility for flight will be influenced by features built for the original context” (*Ibid*.). This diversion through Nietzsche underlines the fact that the historical constraints that have come to crystallise in evolution also constitute reserves of potentialities. Gould refers to the remarkable case of overloading found in the black heron *Egretta Ardesiaca*, which uses its wings to shade the shallow water of its habitat, “thus providing a clear view of available prey” (*Ibid.,* p. 1225). It is implausible that the wings originally evolved for the sole purpose of shading so that the herons could better see their prey. On the other hand, it is probable that wings evolved in the context of the advantage that flight presented for these animals, and that subsequently this structural adaptation may have taken on another possible meaning: that of shading to find prey. But Gould goes even further back: in fact, it is likely that “feathers were initially coopted for flight in a much older functional shift, a defining transition in avian phylogeny from a different initial role, perhaps in thermoregulation” (he refers to Kingsolver & Koehl, 1985; Gould, 2002, p. 1226). This example enriches the aptive triangle *by an excess at the level of the functional vertex, which would come from the creative circularity of the different constraint vertices* (Fig. 3). The causality is thus less rectilinear than circular. And there would be a sort of indefinite accumulation at the functional level, an accumulation which is no longer so much structural (even if it is accompanied by phenotypic changes) as semantic.

### A continual reinterpretation

There is a causal circularity coming from the perpetual reinterpretation of the constraints by the organisms which make them true evolutionary norms. From this perspective, it should be underlined that some constraints did *not* emerge from the functional vertex: there are some actual aptive features which were originally non-aptations. Their evolutionary meaning changed in the course of history. This is the case of the fontanelles we mentioned. This also the case of the repetitive DNA that Gould and Vrba describe as “nonapted features, available for cooptation later, but not serving any direct function at the moment. When coopted, they will be exaptations in their new role (with secondary adaptive modification if altered)” (1982, p. 11). Another example provided by Gould (2002, pp. 1282–1284) which will enable us to apprehend the evolutionary dynamics of these constraints, this time on an ecological scale, is that of *Linepithema humile*. In Argentina, these ants split into colonies of genetically closer individuals, colonies that compete with each other, with the result that the entire species remains at relatively low population density levels. This is not the case in California. The population of ancestors of the ants that invaded California presented a genetic bottleneck (the population probably came from a single indigenous colony in Argentina). Being all very close genetically, they formed a single large colony of ants which quickly invaded California, their population not being regulated by inter-colonial conflicts. Their ecological success in California paradoxically stems from the disappearance of an adaptation (colony recognition) induced by the loss of genetic diversity. Hence, the constraint represented by the loss of diversity proved adaptive in this particular context and transformed the Californian biotope. It should be added that this feature could also prove to be “pathological” in the event of sudden environmental changes, since the lack of competition could again lead to a genetic drift that would either lead to a situation like that in Argentina, or to extinction.

These examples show the creativity of constraints (3) through their perpetual reinterpretation. They reveal the way in which these reinterpretations emerge from evolution itself, as a historical process (2), while at the same time channelling that same process (1). But it is (4) that is crucial to understanding the modality of their causal power. Gould often refers to constraints as forming “reservoirs of potential” (2002, p. 49) and as generating evolvability (*Ibid.*, pp. 1270–1295). He writes about the genetic development pathways: “the constraints of these rules have provided more flexibility in their fecund channels than limitations through their 'forbidden places'” (*Ibid.,* p. 1272). But how do these constraints orient evolution towards non-determined possibilities? How do they induce evolutionary change? By what causality do they channel evolution? There are two indissociable questions here: what is the exact modality of their causal power? And where do they get this causal power from? This is where we think it is crucial to understand evolutionary constraints as norms: these two questions are resolved if the power of these constraints is understood as normative.

### Trying to think modality beyond Aristotelian categories

In Aristotle (2017, pp. 12, 21a35—13, 23b27), there are four fundamental modalities. The possible (that which does not imply contradiction and can therefore be true) is the contradiction of the impossible (that which implies contradiction, i.e., that which cannot be true); the necessary (that which cannot be otherwise than it is and therefore cannot not be true) is the contradiction of the contingent (that which could have been different from what it is; its contrary was also possible, i.e., it can be false). These four modalities imply a pre-determined space of possibilities, and hence appear insufficient for thinking of the causal force of evolutionary constraints, and of their potential. Most physical laws entail a necessity: they indicate that, in a given situation, if the conditions are met, the effect will occur. But biological constraints do not necessitate as physical laws do. Their historicity and the multiplicity of singular processes that characterise the situations in which they are actualised make them shifting norms that do not display a predictive uniformity. And yet, if they widen the possibilities of evolution, they allow the organisation of biological phenomena, in so far as evolution is not a process that would tend more and more towards randomness. This is why their normative power cannot be considered as part of the category of possibility either. They orient evolution in such a way that it does not realise just any possibility, but some possibilities rather than others: the most functional and relevant ones, the possibilities leading to an increasingly organised evolution.

Mumford and Anjum seek a third modal path between necessity (and its contradictory: contingency) and possibility (and its contradictory: impossibility), because these categories do not allow for an account of the canalising power of processes (2018a, pp. 31–35) which they speak of in terms of tendency. Processes *tend* in certain directions, without these directions being necessitated, nor having the status of mere possibilities. The modal status of the tendency would be irreducible to the traditional modalities and would lie somewhere between pure necessity and pure contingency (Mumford & Anjum, 2011, p. viii). They call this modality *dispositional* and ground it on a metaphysical conception of reality that conceives of all causality in terms of process (*Ibid.,* p. ix).^2^ In *Getting causes from powers*, the dispositional modality is described as follows: given a cause A, the effect B is not just one possibility among others; A *tends* to manifest B if nothing prevents it. However, this formulation fails to really break out of the traditional logic because it could be connected to a sense of contingency already present in Aristotle: ὡς ἐπὶ τὸ πολύ (what most often occurs, 2009, I 3, 25b14 and I 13, 32b7) and which is statistic. Mumford and Anjum write that the dispositions are “disposed to happen in a non-negligible probability” (2011, p. 181). Moreover, it seems that dispositional modality is not irreducible to necessity, since it could be described in terms of conditional necessity (the expected effect occurs if other causes do not interfere). According to Sinclair (2019), in order to account for this *sui generis* modality, one would have to go beyond external manifestation and say something about the internal force, insofar as it may not come to manifestation. This is what Mumford and Anjum propose in *What tends to be* when they argue that a tendency is an internal force that may produce an effect without requiring it but may also not come to its manifestation even though there is nothing to prevent it (2018a, pp. 17–18). The force or power in its manifestation is intrinsically a function of tendency rather than necessity. It is the power of a causal process constituted by its temporal thickness: it takes time to unfold, a time during which “some properties will be lost, and new properties and new interactions might be introduced” (*Ibid.*, p. 80). Sinclair (2019) tends to account for this last point by calling on the internal experience of inclination that would allow us to think concretely of dispositional modality by revealing the efficacy of the very duration of the process. It is also this processual dimension that Mumford and Anjum emphasise to conceive causality in biology, a realm characterised by perpetual change and activity (2018b). They give the example of pregnancy: it can be thought that it goes from cause (the sexual act) to effect (the birth of a child). However, pregnancy is not a linear process going from reproduction to birth; many other processes are at stake which interact with and even contribute to this primary process: bodily, physiological, hormonal changes…

### Normative modality and biological practices

But this approach seems insufficient for biological evolution, in two respects. On the one hand, it does not account for the reconfiguration of the space of possibilities specific to biological evolution. The authors conceive the dispositional modality as applying to any phenomenon. They underline that there are degrees of possibilities, which seem to be linked to probability. Yet, probability is a measure on a given space of possibilities. Thus, they do not address the truly innovative nature of the possibilities created during evolution. The authors investigate the causal link between A and its typical manifestation B. But in evolution, B is not predetermined, and is therefore not predictable no matter how much information is available about A. It is no coincidence that the biological example given by Mumford and Anjum is not borrowed from evolutionary biology. In evolution there are no typical manifestations, only special cases. As Longo puts it «biological events are rare […] in view of their biological specificity or historicity: each event is *individually* rare, even if this *type* of events happens continually in evolution and contribute to the construction of *all* phylogenetic paths” (2018, p. 469). On the other hand, the authors underline the close link between the manifestation of the effect and the continuity of the process that causes it, but do not account for the fact that, in evolution, the causality displays a creative circularity: the evolutionary norms, are both produced by and producers of the evolutionary process. They indicate that what accounts for dispositionality in biology is the fact that life is made up of activities, but do not directly link these activities with their conception of causality.

We believe, on the contrary, that these activities must be at the centre of a conception of causality in evolution, since it is they that account for the creativity of evolutionary constraints as well as their specific causality. It is because constraints arise from interactions that are woven by agents, and because, from this point of view, they constitute real biological *norms*, that they have the power to canalise evolution. To understand the causality of constraints in evolution, it is therefore necessary to take into consideration the agency of living beings. For it is living beings that internalise and manifest them through practices, i.e., activities that are embedded in and transform their living conditions and thus the rest of evolution. This is why, to envision this driving role of organisms in evolution, Walsh proposes an agent theory. For ecological constraints, for example, to have a truly positive action, they must form opportunities for organisms. Hence, the latter must be true agents, i.e., beings capable of experiencing these conditions as affordances and of responding to them through practices likely to transform these very conditions (Walsh, 2015, p. 163). The living themselves actively constitute the constraints to which they are subject, through their agency (*Ibid.,* p. 173). Thus, the power of the evolutionary constraints comes from their normative part, from the way in which living beings internalise them in activities that create new constraints that can transform evolutionary history. If we look back at the example of the functional overloading of the black heron’s wings, the morphological constraint (the feathers) resulting from the adaptation of thermoregulation constituted a crucial opportunity since it enabled the heron to fly. The practice of flight by the heron led to a selection such that the proto-wings became real wings: this practice was actualised in a morphological constraint, underpinned by genetic processes. And in return, this constraint allowed the emergence of a further new practice (and therefore also a new function) through the use of the parasol wing. The creative circularity of evolutionary constraints becomes illuminating when they are understood as *norms whose multiple meanings arise from the concrete practices of organisms conceived as biological agents*. Similarly for the diverse interactions of *Wolbachia*: it is within each interaction, in the way it is concretely tied up by the agents involved, that evolvability is played out. Neither the historical, morphological and ecological data nor the imperative of adaptation *determine* the directions taken by evolution. Instead, these constraints act as norms that living beings internalise into biological activities canalising future evolution*.* Let us emphasise that internalisation here is not psychological nor subjective: living beings are not necessarily cognitive agents, but they are agents insofar as they experience these biological norms in the particularity of their situation and transform them by their likewise particular practice. The changes in meaning of the black heron’s wing, or of the interaction of host and parasite, do not require a passage through conscious subjectivity, but only *a passage through the activity of the living as historical interactions that constantly transform the constraints they inherit into new possibilities*.

This leads us to the first point that the dispositional modality failed to account for: the change in the space of possibilities, and thus the unpredictability of evolution. The various consequences of the presence of *Wolbachia* on the evolution of its host reveal the importance of the internalisation of norms in interactions which are always particular cases. The biological norm is always local and transitory, i.e., specific to a time and place in evolutionary history. And it is within these “rare” conditions—in the sense given by Longo (2018): specific, historical—that living beings experience these norms through practices that are also rare. Therefore, it is because evolutionary constraints constitute norms for living beings that they can be truly innovative: they owe their power to the way organisms internalise them. They arise from the evolutionary process as it is shaped by the activities and practices of living beings, but they also channel this process, and even generate its future possibilities, *through internalisation, i.e.,* the way in which living beings experience these norms and actualise them through practices in the specificity of their unique situation. *It is precisely because these norms are internalised in the practices of particular agents in specific situations that evolution is truly unpredictable (the phase space is perpetually changing).*

The outcome B is not predictable from the norm A, because the very possibility of B emerges from the way particular agents internalise A through an equally particular or rare practice C. It is from this internalisation that biological constraints derive their power: they become norms for evolution.

## Conclusion

Our aim was to clarify the causal power of biological constraints on the evolution of biodiversity. We argued that this power became illuminating if thought of in terms of normativity. We began with a critical moment aimed at identifying the inadequacies of the explanations traditionally proposed to account for regularities in biology. Moving away from these nomological, often monolithic, and in any case static conceptions of evolution, we proposed an analysis of the multiple constraints that canalise the forms taken by biodiversity. We highlighted that these constraints were historical, and that this history was woven by the interactions of living beings. We then studied these interactions to show that they had a normative dimension: they may be vital or fatal for the survival of organisms, and they redefine their conditions of existence. This led us to reconceptualise biological constraints in evolution in terms of evolutionary *norms,* embracing four characteristics. They appear as binding conditions for biological evolution (1), deriving from the evolutionary process itself, while channelling it and opening its future possibilities (2). These possibilities are not predetermined in a predefined phase space (3) but emerge from the very way in which living beings internalise these norms in practices specific to their particular situation (4). Finally, we have shown that conceiving biological constraints in evolution as evolutionary norms allows us to shed light on the modality of their causal power, inadequately captured by the categories of traditional logic inherited from Aristotle, while accounting for the nature of the entities at stake: biological agents. This concept of normativity is not intended to replace the existing work on constraints, nor the work on process biology, but it is meant to elucidate the role of constraints on the directions taken by biological evolution, bringing forward the agency of living beings.

## Footnotes

1 Insofar as he wants to distinguish these constraints from the “classical”, *i.e.,* functionalist, causes of Darwinism, he restricts his definition of constraints to morphological structuralism.

2 It should be stressed that the modality they propose is rooted in a metaphysics of powers that presuppose a non-necessitarian conception of causality *in general,* and not just for certain processes. We do not wish to get into such a metaphysical consideration but use their model to consider the modality of biological phenomena, insofar as it allows us to overcome Aristotelian logic by thinking causality in a processual way.
